# Animal source food intake and association with blood cholesterol, glycerophospholipids and sphingolipids in a northern Swedish population

**DOI:** 10.3402/ijch.v72i0.21162

**Published:** 2013-08-05

**Authors:** Wilmar Igl, Afaf Kamal-Eldin, Åsa Johansson, Gerhard Liebisch, Carsten Gnewuch, Gerd Schmitz, Ulf Gyllensten

**Affiliations:** 1 Department of Immunology, Genetics and Pathology, Rudbeck Laboratory, University of Uppsala, Uppsala, Sweden; 2 Department of Food Science, Faculty of Food and Agriculture, United Arab Emirates University, Al-Ain, UAE; 3 Institute for Clinical Chemistry and Laboratory Medicine, Regensburg University Medical Center, Regensburg, Germany

**Keywords:** epidemiology, nutrition, animal source foods, game, lipids, cholesterol, phospholipids, sphingolipids

## Abstract

**Background:**

The high intake of game meat in populations with a subsistence-based diet may affect their blood lipids and health status.

**Objective:**

To examine the association between diet and circulating levels of blood lipid levels in a northern Swedish population.

**Study design:**

We compared a group with traditional lifestyle (TLS) based on reindeer herding (TLS group) with those from the same area with a non-traditional lifestyle (NTLS) typical of more industrialized regions of Sweden (NTLS group). The analysis was based on self-reported intake of animal source food (i.e. non-game meat, game meat, fish, dairy products and eggs) and the serum blood level of a number of lipids [total cholesterol (TC), low-density lipoprotein cholesterol (LDL), high-density lipoprotein cholesterol (HDL), triglycerides (TG), glycerophospholipids and sphingolipids].

**Results:**

The TLS group had higher cholesterol, LDL and HDL levels than the reference group. Of the TLS group, 65% had cholesterol levels above the threshold for increased risk of coronary heart disease (≥240 mg/dl), as compared to 38% of the NTLS group. Self-reported consumption of game meat was positively associated with TC and LDL.

**Conclusions:**

The high game meat consumption of the TLS group is associated with increased cholesterol levels. High intake of animal protein and fat and low fibre is known to increase the risk of cardiovascular disease, but other studies of the TLS in northern Sweden have shown comparable incidences of cardiovascular disease to the reference (NTLS) group from the same geographical area. This indicates that factors other than TC influence disease risk. One such possible factor is dietary phospholipids, which are also found in high amounts specifically in game meat and have been shown to inhibit cholesterol absorption.

Intake of high-energy food and physical inactivity are both associated with increased obesity and related human disorders, such as diabetes and cardiovascular disease. In addition to obesity, a number of diet-related risk factors are known to influence disease risk, such as blood lipids, particularly high levels of cholesterol and saturated fatty acids. High consumption of animal-derived foods rich in these lipids, such as red meat, milk, dairy products and eggs have been regarded as potentially contributing to disease risk (1). Some studies have yielded differing results, possibly due to counteracting effects of other components in these foods, such as calcium and conjugated linoleic acid in milk and dairy products, and phosphatidylcholine (PC) and other phospholipids in egg yolks (2).

Human circumpolar populations are going through a change from a subsistence-based lifestyle to the lifestyle in more industrialized regions. In order to address the adverse effects of this transition, it is important to understand the long-term effect of these lifestyle changes on the health of native populations. In this study, we examined the associations between dietary intake and the levels of circulating levels of lipids in regions where one part of the population leads a subsistence-based lifestyle based on reindeer herding (3,4), while the other part has a lifestyle similar to that of industrialized areas of Western Europe.

## Materials and methods

### Study population

The study is located in the parish of Karesuando in the northernmost part of Sweden and is part of the Northern Swedish Population Health Study (NSPHS). The NSPHS was approved by the regional ethics committee at the University of Uppsala (Regionala Etikprövningsnämnden, Uppsala, Dnr 2005:325). The study adheres to the principles of the Declaration of Helsinki and Code of Federal Regulations of the USA, Part 46, Protection of Human Subjects and all participants signed a written informed consent.

Socio-demography (e.g. sex, age and occupation) and body size measures (e.g. height, weight and body mass index) were assessed, and the participants were divided based on self-reported information on sex and occupation. In the following, these occupations/lifestyles were referred to as traditional lifestyle (TLS) versus non-traditional lifestyle (NTLS).

### Diet

Data were collected with a food frequency questionnaire based on the Northern Sweden 84-item Food Frequency Questionnaire (NoS-84-FFQ), validated and applied within the Västerbotten Intervention Programme (5). For each food item, we calculated daily intake in grams per day as a standardized unit of measurement and aggregated the items to food categories, such as non-game meat, game meat, fish, dairy products and eggs. The average, unweighted nutrient contents (e.g. fat, protein, carbohydrates and fibre) of the examined food categories were calculated using the food database of the Swedish Food Administration (6). Since several items were added to the NoS-84-FFQ questionnaire on foods specific for the lifestyle in this geographic region, in particular on game consumption (reindeer, moose), we evaluated the construct validity (known-groups validity) of the items on game consumption. We compared the groups traditional (N=94) versus non-traditional lifestyle (N=505). We observed highly significant effects in men (effect size or ES=(M_TLS_−M_MLS_)/SD_pooled_=1.15, p=2.3×10^−5^) and women (ES=1.25, p=8.2×10^−4^) in the expected direction corresponding to a three times higher consumption of game intake in those with a traditional lifestyle.

### Physical activity

Two self-report scales were used to measure physical activity at work and at leisure. The Work Activity Scale (WAS, 6 items) addresses occupational physical activities, such as sitting, standing, walking, lifting and general indicators of physical activity. The Leisure Activity Scale (LAS, 4 items) asks for typical free-time activities such as walking, cycling, other sporting activities and sweating as a general indicator of physical activity. Both scales showed satisfying internal consistency with Cronbach's α(WAS)=0.73 and Cronbach's α(LAS)=0.70.

### Circulating lipids

Total cholesterol (TC), low-density lipoprotein cholesterol (LDL), high-density lipoprotein cholesterol (HDL), and triglycerides (TG) in blood serum were quantified by enzymatic photometric assays using an ADVIA1650 clinical chemistry analyzer (Siemens Healthcare Diagnostics GmbH, Eschborn, Germany) at the Institute for Clinical Chemistry and Laboratory Medicine, Regensburg University Medical Center, Germany. Glycerophospholipids and sphingolipids in the blood plasma were quantified by electrospray ionization tandem mass spectrometry (ESI-MS/MS) in positive ion mode as described previously (7,8). A neutral loss scan of m/z 141 was used for phosphatidylethanolamine (PE) (9) and PE-based plasmalogens (PE-pls) as described (10). Absolute levels of classical lipids (TC, LDL, HDL, and TG) were measured in mg/dl and levels of glycerophospholipids and sphingolipids in µMol/l.

### Statistical analysis

Distribution statistics for diets, physical activity and circulating lipids were based on untransformed values. Association statistics used transformed values adjusted for sex, age, age×age and total energy intake by linear regression models. To compute inferential statistics (e.g. p-values), the adjusted values were converted to normal distribution by inverse-normal transformation. A general linear mixed effects model was used to adjust the outcomes for pedigree structure and covariates (11,12). For descriptive, comparative results for relevant subgroups in our sample, we calculated sample size independent of effect size measures (ES=(M_TLS_−M_MLS_)/SD_pooled_) to evaluate the strength of the effects for quantitative traits, using established thresholds. Nominal statistical significance was based on a local type I error of α=0.05. We addressed the issue of multiple testing by reporting all conducted statistical tests as recommended.

## Results

### Diet and body measures

There was no difference between the TLS and NTLS groups in weight, but TLS women were shorter (p=0.0021) (Table I). TLS men were of the same age as the NTLS men, while TLS women were on average 9 years older (p=0.0081) than the sex-matched NTLS women.

**Table I:** Body measures, food intake, nutrient intake and physical activity in men and women with a traditional lifestyle (TLS) or a lifestyle similar to that of industrialized region (NTLS)

	Men				Women				Men and women (TLS vs. NTLS)	
			
	TLS (N=56)	NTLS (N=224)	Effect size^a^	p^b^	TLS (N=38)	NTLS (N=281)	Effect size^a^	p^b^	Effect size^a^	p^b^
a) Body measures; M(SD)										
Age (years)	51.6 (15.9)	50.5 (20.0)	+0.06	0.12	56.8 (15.4)	48.0 (19.6)	+0.46	8.1E−3**	+0.24	4.5E−3**
Weight (kg)	74.7 (12.5)	81.0 (14.4)	−0.45	0.63	64.3 (10.6)	66.3 (13.1)	−0.15	0.70	−0.15	0.73
Height (cm)	165.8 (8.4)	172.5 (6.8)	−0.93	8.1E−2	152.4 (6.5)	159.2 (6.7)	−1.02	2.1E−3**	−0.49	0.11
BMI (kg/m^2^)	27.0 (3.2)	27.2 (4.7)	−0.04	0.54	27.7 (4.2)	26.2 (5.0)	+0.29	6.2E−2	+0.13	0.42
b) Self-reported food intake; M(SD)										
Meat, total (g/day)	226.4 (190.6)	144.1 (108.3)	+0.64	2.9E−3**	182.0 (91.0)	131.8 (85.0)	+0.59	2.2E−3**	+0.66	1.4E−4***
Meat, non-game (g/day)	36.2 (32.7)	83.4 (56.2)	−0.90	2.2E−7***	42.1 (35.2)	75.4 (68.0)	−0.51	4.8E−2*	−0.68	1.2E−4***
Meat, game (g/day)	190.2 (181.8)	60.7 (88.6)	+1.15	2.3E−5***	139.9 (93.0)	56.5 (62.9)	+1.25	8.2E−4***	+1.21	1.0E−7***
Fish (g/day)	18.3 (19.3)	24.3 (26.5)	−0.24	0.17	18.3 (20.9)	24.1 (26.5)	−0.22	0.12	−0.23	6.3E−5***
Milk products (g/day)	282.3 (269.1)	542.7 (443.2)	−0.63	2.6E−3***	360.2 (277.0)	403.0 (392.2)	−0.11	0.18	−0.38	4.8E−2*
Eggs (g/day)	4.2 (4.3)	5.7 (6.7)	−0.24	0.17	6.4 (8.2)	5.9 (8.7)	+0.07	0.32	−0.16	0.13
c) Self-reported nutrient intake; M(SD)										
Fibre (g/day)	19.9 (15.0)	17.3 (8.4)	+0.26	0.23	22.8 (10.9)	23.6 (13.6)	−0.06	0.21	+0.03	0.12
Carbohydrates (g/day)	235.2 (211.9)	220.4 (97.3)	+0.12	0.20	242.0 (141.9)	252.4 (132.2)	−0.08	0.18	+0.00	0.27
Protein (g/day)	109.6 (82.1)	85.5 (37.9)	+0.49	0.11	96.5 (38.2)	87.1 (38.9)	+0.24	0.17	+0.40	1.5E−4***
Fat (g/day)	57.2 (30.5)	61.1 (24.9)	−0.15	0.12	51.3 (23.0)	55.4 (23.6)	−0.17	0.20	−0.13	2.6E−2*
Cholesterol (mg/day)	374.5 (262.7)	276.9 (141.7)	+0.57	2.18E−4***	283.9 (123.8)	251.2 (107.6)	+0.30	0.13	+0.52	6.2E−4***
Alcohol (g/day)	0.96 (1.53)	1.70 (2.49)	−0.32	0.15	0.22 (0.52)	0.90 (1.75)	−0.41	4.8E−3**	−0.30	3.8E−4***
Energy (kjoule/day)	8,169 (5,816)	7,653 (2,876)	+0.14	0.25	7,845 (3,513)	8,038 (3,450)	−0.06	0.28	+0.05	0.43
d) Self-reported physical activity; M(SD)										
Work activity^c^	3.21 (0.63)	2.96 (0.67)	+0.43	5.2E−2	2.82 (0.61)	2.94 (0.64)	−0.19	0.12	+0.15	7.3E−2
Freetime activity^c^	2.43 (0.79)	2.76 (0.75)	−0.38	4.2E−2*	2.39 (0.96)	2.89 (0.82)	−0.60	7.3E−3**	−0.52	5.0E−3**

a Effect sizes (ES) are written out as untransformed, original values: ES=(M_TLS_−M_MLS_)/SD_pooled_ for clarity.

b p-values are based on the inverse-normally transformed residuals (see Methods).

c Frequency of physical activity: 1=“never” to 5=“very often”.

* p=(0.01; 0.05)=(1.0E−2; 5.0E−2)

** p=(0.001; 0.01)=(1.0E−3; 1.0E−2)

*** p=(0.00; 0.001)=(0.0; 1.0E−3).

TLS men and women consumed on average 57 and 38% more meat, respectively, compared to the same-sex NTLS group (men: 226.4 vs. 144.1 g/day, p=2.86×10^−3^; women: 182.0 vs. 131.8 g/day, p=2.2×10^−3^; p_total_=1.4×10^−4^). The intake of game meat was about 213% higher for TLS men and 148% higher for TLS women than for the corresponding NTLS group (men: 190.2 vs. 60.7 g/day, p=2.3×10^−5^; women: 139.9 vs. 56.5 g/day, p=8.2×10^−4^; p_total_=1.0×10^−7^), whereas the intake of non-game meat was about 50% less in TLS (men: 36.2 vs. 83.4 g/day, p=2.2×10^−7^; women: 42.1 vs. 75.4 g/day, p=0.048; p_total_=1.2×10^−4^). The TLS group consumed less fish (men: 18.3 vs. 24.3 g/day, p>0.05; women: 18.3 vs. 24.1 g/day, p>0.05; p_total_=6.3×10^−5^) and milk products than the NTLS groups (men: 282.3 vs. 542.7 g/day, p=0.0026; women: 360.2 vs. 403.0, p>0.05; p_total_=0.048). Nominally significant differences between groups indicate a higher protein (p=1.54×10^−4^), lower fat (p=0.026) and lower alcohol (p=3.8×10^−4^) content for the TLS group. No difference was seen between groups in physical activity at work (p>0.05), but the NTLS group had higher activity during leisure time (p=0.042). For women, the two groups showed similar physically active at work (p>0.05), but the NTLS showed significantly higher activity during free time (p=0.0073) (Table I).

### Circulating lipids

Higher levels of TC, LDL and HDL were found in the TLS group (TC−men: 246 vs. 226 mg/dl, p>0.05; women: 246 vs. 230 mg/dl, p>0.05; p_total_=7.8×10^−3^, LDL−men: 154 mg/dl vs. 138 mg/dl, p=0.041; women: 137 vs. 146 mg/dl, p>0.05; p_total_=0.029 and HDL−men: 63 vs. 55 mg/dl, p=0.012; women: 69 vs. 67 mg/dl, p>0.05; p_total_=7.1×10^−3^). Mean TC levels in the NTLS group (226 and 230 mg/dl for men and women, respectively) were close to the threshold for increased risk of cardiovascular disease (≥240 mg/dl), while the TC levels in the TLS group (246 mg/dl for both sexes) were above the threshold. In total, 65% of the participants in the TLS group fall into the high-risk category, as compared to only 38% of the NTLS group (Table II).

**Table II:** Levels of circulating lipids in men and women with a traditional lifestyle (TLS) and a lifestyle similar to that of industrialized region (NTLS)

	Men				Women				Men and women (TLS vs. NTLS)	
			
Lipid category	TLS (N=56)	NTLS (N=224)	Effect size^a^	p^b^	TLS (N=38)	NTLS (N=281)	Effect size^a^	p^b^	Effect size^a^	p^b^
a) Classical lipids (mg/dl, blood serum); M(SD)										
Total cholesterol (TC)	246 (44)	226 (49)	+0.41	0.12	246 (45)	230 (51)	+0.32	0.13	+0.36	7.8E−3**
LDL cholesterol (LDL)	154 (42)	138 (39)	+0.39	4.1E−2*	146 (39)	137 (42)	+0.22	0.27	+0.33	2.9E−2*
HDL cholesterol (HDL)	63 (17)	55 (12)	+0.59	1.2E−2*	69 (14)	67 (16)	+0.10	0.80	+0.22	7.1E−3**
Triglycerides (TG)	205 (96)	241 (169)	−0.23	0.67	188 (107)	173 (99)	+0.16	0.13	−0.03	0.40
b) Glycerophospholipids (µMol/l, blood plasma); M(SD)										
Glycerophospholipids (GP), total	2,795 (491)	2,627 (517)	+0.33	6.4E−2	2,799 (501)	2,685 (525)	+0.22	0.17	+0.27	4.4E−2*
Phosphatidylcholines (PCs)	2,368 (428)	2,225 (463)	+0.32	3.6E−2*	2,429 (1,394)	2,312 (485)	+0.24	0.14	+0.25	5.2E−2
Lysophosphatidylcholines (LPCs)	313 (64)	319 (74)	−0.09	0.28	275 (59)	293 (75)	−0.25	0.14	−0.10	0.22
Phosphatidylethanolamines (PEs)	27.5 (9.8)	21.0 (7.9)	+0.78	1.5E−3**	23.3 (7.0)	19.9 (7.0)	+0.48	0.19	+0.70	5.6E−8***
PE-based plasmalogens (PE-pls)	86.1 (33.8)	62.5 (25.0)	+0.88	3.1E−4***	72.3 (23.8)	60.2 (22.4)	+0.54	0.24	+0.78	4.0E−9***
c) Sphingolipids (µMol/l, blood plasma); M(SD)										
Sphingolipids, total	565 (111)	499 (114)	+0.58	0.14	612 (121)	554 (117)	+0.49	5.0E−2***	+0.46	4.1E−5***
Sphingomyelins (SMs)	555 (110)	490 (113)	+0.58	2.2E−2*	601 (119)	545 (116)	+0.49	5.6E−2	+0.46	4.2E−5***
Ceramides (CERs)	10.3 (2.3)	9.5 (2.5)	+0.30	3.7E−2*	10.6 (2.7)	9.2 (2.4)	+0.58	2.0E−2*	+0.43	3.7E−3**

a Effect sizes (ES) are written out as untransformed, original values: ES=(M_TLS_−M_MLS_)/SD_pooled_ for clarity.

b p-values are based on the inverse-normally transformed residuals (see Methods).

* p=(0.01; 0.05)=(1.0E−2; 5.0E−2)

** p=(0.001; 0.01)=(1.0E−3; 1.0E−2)

*** p=(0.00; 0.001)=(0.0; 1.0E−3).

For the phospholipids (PLs), large differences were found between the TLS and NTLS groups for the PEs (men: 27.5 vs. 21.0 µMol/l, p=0.0015; women: 23.3 vs. 20.0 µMol/l, p>0.05) and PE-pls (men: 86.1 vs. 62.5 µMol/l, p=3.1×10^−4^; women: 72.3 vs. 60.2 µMol/l µMol/l, p>0.05), while less so for PCs (men: 2,368 vs. 2,225 µMol/l, p=0.036; women: 2,429 vs. 2,312 µMol/l, p>0.05) and no difference was seen for lysophosphatidylcholines (LPCs) (Table II). Nominally significant differences were found between the TLS and NTLS groups for PEs (p_total_=5.6×10^−8^), PE-pls (p_total_=4.0×10^−9^), and total GPs (p_total_=0.044) (Table II).

Total sphingolipids (SLs) also differed between TLS and NTLS women (612 vs. 554 µMol/l, p=0.05), but not between men (565 vs. 499 µMol/l, p>0.05). Differential effects were found for sphingomyelin (SMs) levels in men (555 vs. 490 µMol/l, p=0.022), but not in women (601 vs. 545 µMol/l, p>0.05). However, both TLS men and women showed higher levels of ceramides (CERs) compared to NTLS individuals (men: 10.3 vs. 9.5 µMol/l, p=0.037; women: 10.6 µMol/l vs. 9.2 µMol/l, p=0.020). Significant differences in SMs were also seen between the TLS and NTLS groups (p=4.2×10^−5^), CERs (p=3.7×10^−3^) and total SLs (p=4.1×10^−5^) in the overall sample (Table II).

### Food intake and circulating lipids

Self-reported intakes of animal source food categories were associated with measured levels of TC, LDL, HDL and TG, as well as with the PC, LPC, PE, PL, SM and CER lipid classes (Supplementary Table I). Daily intake of game meat was positively associated with TC (r=0.11, p=0.016) and HDL (r=0.11, p=0.020) and non-game meat negatively associated with TC (r=−0.09, p=0.045) and TG (r=−0.11, p=0.012). Significant correlations were observed between game meat intake and PCs (r=0.12, p=0.011), PEs (r=0.25, p=1.6×10^−8^) and in particular PE-pls (r=0.24, p=2.5×10^−8^). Also, fish intake was associated with PEs (r=0.12, p=1.5×10^−3^) and PE-pls (r=0.11, p=3.7×10^−3^). Consumption of milk products was negatively associated with levels of LPCs, PEs and PE-pls (all r=−0.11, p≤7.6×10^−3^).

### Food intake and fatty acids in polar lipids

Game meat was associated with all four PC subcategories ranging from r=0.09 (p=0.041) for polyunsaturated to r=0.21 (p=7×10^−6^) for alkylated PCs. Fish consumption lowered the level of monounsaturated LPCs (r=−0.11, p=0.0099), and this was the only LPC subcategory showing an association (Supplementary Table II). High intake of non-game meat was related to lower levels of mono- and polyunsaturated PEs [r=(−0.14; −0.12), p=(0.0010;0.0060)] but not saturated PEs. Game intake strongly increased levels of all subcategories of PE-based Plasmalogenes [(PE p16:0/x (r=0.25, p=1.6×10^−8^), PE p18:1/x (r=0.13, p=4.2×10^−3^) and PE p18:0/x (r=0.26, p=2.6×10^−9^)]. Fish intake showed similar effects [r=(0.08;0.12), p=(5.0×10^−5^; 0.029)]. Only consumption of game meat increased levels of dihydro SMs and total (r=0.10; p=0.020), saturated and glycosylated [all r=0.09, p=(0.040;0.047)] CERs.

## Discussion

We have examined the differences in body measures, animal-derived food product intake and blood lipids in a population living above the Arctic Circle in northern Sweden, where one part of the population leads a traditional lifestyle (TLS) based on reindeer herding, while the other part has a lifestyle similar to that of the industrialized part of Sweden and other western European countries (NTLS). The TLS group consumed more game meat and less milk products and alcohol compared to the group with a non-traditional lifestyle. Game meat also has the highest content of protein (27.1%), followed by fish (19.1%), non-game meat (17.3%), eggs (12.6%) and milk (7.3%). The cholesterol content is highest in fish (814 mg/kg), followed by game meat (758 mg/kg), non-game meat (605 mg/kg), eggs (417 mg/kg) and milk (228 mg/kg). Both men and women in the TLS group showed higher TC, LDL and HDL levels than the NTLS group (Table II), which is likely to reflect the high intake of game meat (Table I). TC levels >240 mg/dl and LDL >160 mg/dl are regarded as risk factors for coronary heart disease. A combination of high levels of animal protein and fat and low levels of fibre, such as seen for the TLS group, are known to increase the risk of cardiovascular disease (13). Nevertheless, reindeer herding Sami of northern Sweden have a comparable incidence of cardiovascular disease to other populations in the same geographical area, indicating that other factors are affecting their disease risk (14,15).

One factor that might modulate the effect of cholesterol is polar lipids, such as glycerophospholipids. The addition of PC prolongs the nucleation time, increases cholesterol solubility, and prevents cholesterol gallstones (16). Egg yolk lecithin, which contains PE and PC, decreases total serum cholesterol and apo A-I, and increases serum apo B, the excretion of faecal cholesterol and coprostanol (17). It has also been suggested that dietary PLs inhibit cholesterol absorption (18), which might explain the small differences in cholesterol levels in these populations despite the differences in cholesterol intake. Macronutrients contribute to the metabolic overload, but research on lipid signalling suggests that the quality of both macro- and micronutrients plays an important role (19–21). Sphingolipid- and cholesterol-rich lipid domains embedded in glycerophospholipids play central roles in the assembly of lipid rafts and it is suggested that these are involved in adipocyte physiology, cardiovascular disease and carcinogenesis (22).

The consumption of non-game meat was associated with lower levels of a broad range of the lipids studied, including cholesterol, glycerophospholipids and sphingolipids. This food category seems to be of little importance for most LPCs and PE-pls, but shows negative effects on PCs and PEs. The consumption of game meat was associated with higher levels of a broad range of lipids, including cholesterol, glycerophospholipids and sphingolipids, which are known risk factors for cardiovascular and metabolic disease. In particular, venison elevated levels of polyunsaturated PCs, polyunsaturated, long-chain PEs and a broad spectrum of mono- and polyunsaturated PE-pls. The positive association of game meat with cholesterol levels might appear questionable since game contains much more protein (27.1 vs. 17.3%) and less fat (7.4 vs. 12.8%) compared to non-game meat. However, game meat has higher specific cholesterol content (0.76%) compared to pork or beef (0.60%) (6). The plant sterols found in vegetables also inhibit the intestinal uptake of cholesterol (23). The lower intake of vegetables (3,4) can therefore contribute to higher cholesterol levels in the TLS group. Fish intake strongly increased blood plasma levels of LPCs and PE-pls, which bind elcosapentaenoic acid (EPA) and docosahexaenoic acid (DHA) fatty acids. Associations between fish intake and circulating EPA and DHA fatty acids are well documented. Negative correlations between fish intake and certain lipids might point to certain fatty acids in the glycerophospholipid fraction being used as precursors for conversion into EPA and DHA (24).

The health effects of blood levels of some of the examined lipid species are currently not fully understood. High cholesterol has been shown to increase the risk of cardiovascular disease, while n-3 fatty acids (e.g. EPA and DHA) to decrease risk (25). Of all the food items, only game intake was positively associated with the increased total and LDL values in blood serum. In addition, we observed strong, consistent positive associations between fish intake and blood plasma levels of lipids binding n-3 fatty acids. Since the TLS group consumed more game meat, but less fish than in the NTLS group, their risk for cardiovascular disease may be increased. These results have been corroborated by studies showing increased risk for myocardial infarction in people with a traditional life style (3,26). The health implications of increased blood plasma levels of PLs and SPs are not clear at present. One can assume that not only the altered prevalence of energy-rich lipids and fatty acids in the blood stream, but also the different composition of these lipids can modify cell signalling and risk for metabolic diseases.
